# Response Assessment in Brain Metastases Managed by Stereotactic Radiosurgery: A Reappraisal of the RANO-BM Criteria

**DOI:** 10.3390/curroncol30110679

**Published:** 2023-10-24

**Authors:** Keiss Douri, Christian Iorio-Morin, Rosalie Mercure-Cyr, Gabrielle Figueiredo, Charles Jean Touchette, Laurence Masson-Côté, David Mathieu

**Affiliations:** 1 Division of Neurosurgery, Department of Surgery, Université de Sherbrooke, Centre de Recherche du Centre Hospitalier Universitaire de Sherbrooke, Sherbrooke, QC J1H 5H3, Canada; keiss.douri@usherbrooke.ca (K.D.); christian.iorio-morin@usherbrooke.ca (C.I.-M.); charles.touchette@usherbrooke.ca (C.J.T.); 2 Department of Radiology, Université de Sherbrooke, Centre de Recherche du Centre Hospitalier Universitaire de Sherbrooke, Sherbrooke, QC J1H 5H3, Canada; gabrielle.figueiredo@usherbrooke.ca; 3 Division of Radiation Oncology, Department of Medicine, Université de Sherbrooke, Centre de Recherche du Centre Hospitalier Universitaire de Sherbrooke, Sherbrooke, QC J1H 5H3, Canada; laurence.masson-cote@usherbrooke.ca

**Keywords:** RANO-BM, diameter, progression, response, criteria, brain metastases

## Abstract

Background: Brain metastases (BM) are increasingly being treated using stereotactic radiosurgery (SRS). Standardized response criteria are necessary to improve research and treatment protocols. This study’s goal was to validate the RANO-BM criteria thresholds for tumor progression in a cohort of patients with brain metastases managed using SRS. Methods: We performed a retrospective analysis of patients treated at least twice with SRS for brain metastases. Local progression, as defined by RANO-BM criteria, was compared to our multidisciplinary tumor board’s treatment recommendation. A ROC curve was generated using varying diameter thresholds to assess the sensitivity and specificity of current RANO-BM criteria. Results: 249 metastases in 67 patients were included in the analysis. RANO-BM criteria current progression thresholds yielded a sensitivity of 38%, a specificity of 95%, a positive predictive value of 71%, and a negative predictive value of 84% relative to our tumor board’s treatment recommendation. Modified RANO-BM criteria using absolute diameter differences of 2.5 mm yielded a sensitivity of 83%, a specificity of 87%, a positive predictive value of 67% and a negative predictive value of 94%. Conclusions: Current RANO-BM criteria unreliably identifies clinically relevant tumor progression. The use of absolute diameter differences thresholds appears superior in our BM cohort.

## 1. Introduction

Brain metastases are the most frequently encountered intracranial neoplastic disorders, with an estimated incidence of 150,000–200,000 cases per year, occurring in 20% of patients with cancer [1,2]. Advances in radiotherapy, surgery, chemotherapy and targeted therapy are responsible for better control of the primary disease and longer survival [3]. This improvement in overall survival and increased frequency of cancers responsible for the majority of brain metastases [2], associated with better detection of smaller metastases through magnetic resonance imaging (MRI) [3], have led to significantly higher prevalence of brain metastases.

Brain metastases are associated with significant impairment of the quality of life of cancer patients, leading to symptoms such as headaches, neurocognitive impairment or focal neurological deficits [2]. Palliative treatment, such as stereotactic radiosurgery (SRS), neurosurgical resection or whole-brain radiotherapy (WBRT), is often performed in such patients, and heterogeneous criteria have been used to evaluate response and progression [4], such as RECIST [5], WHO [6], or the Macdonald Criteria [7]. The Response Assessment in Neuro-Oncology Brain Metastases (RANO-BM) working group identified limitations in existing criteria and proposed a more inclusive scale based on RECIST 1.1 and pre-existing RANO high-grade glioma criteria, evaluating not only the radiological features of targeted and non-targeted brain metastases, but also the clinical status, corticosteroid dependency and systemic disease [4]. 

Standardized criteria are beneficial, as they enable appropriate comparisons between different studies and ensure external validity, which would translate to better evidence-based management in current practice. While different thresholds were established by the RANO-BM response criteria to categorize response to treatment, these definitions have never been externally validated since their publication.

The goal of the present study is to validate the use of RANO-BM criteria in order to evaluate the relevance and applicability of such criteria in clinical practice by assessing the sensitivity, specificity, predictive positive value and predictive negative value with current thresholds in a cohort of brain metastases patients managed with SRS in our center.

## 2. Materials and Methods

### 2.1. Participants and Data Collection

We performed a single-center retrospective study of patients actively treated with SRS for brain metastases. This study was approved by our Research Ethics Board (Comité d’éthique de la recherche du CIUSSS de l’Estrie—CHUS, protocol 09-030). Because of the retrospective design and anonymized data collection, no patient consent was necessary. The goal of this study was to compare tumor response to SRS according to RANO-BM criteria to the management prescribed by our multidisciplinary tumor board, which reflected the consensus interpretation of the lesions response, for metastases that were showing an increase in size on follow-up imaging. A cohort was created by screening patients who underwent at least two SRS procedures for intracerebral metastases on the same brain metastasis at the Centre Hospitalier Universitaire de Sherbrooke over a nine-year period, between 2007 and 2016. This allowed selection of patients with, in most cases, multiple metastases, among which at least one presented tumoral progression that warranted retreatment with SRS. This series consequently provided a balanced range of complete responses, partial responses, stable metastatic disease and progressive disease. Only the period between the two SRS procedures was assessed, as it was an active treatment period for the patient in which any significant progression of the disease would have been addressed. The follow-ups after the last SRS treatment were excluded from this study, as they could correspond to palliative care, in which even a significant progression would not be treated, as no intervention could be indicated in the absence of symptoms.

Patient data were collected from their electronic medical record, including age at diagnosis of intracerebral metastases, sex, primary cancer histology, primary cancer surgery, radiotherapy, chemotherapy or targeted therapy before SRS, time between SRS and follow-up, time between both SRS procedures, and metastases diameter before SRS and during follow-up. Extra-axial metastases were excluded from this study. Metastases with a cystic component were excluded from this study due to their variable behavior in response to treatment, and hemorrhagic metastases were also excluded due to diameter changes related to the hematoma resolution.

The outcome of each lesion (progression or non-progression) was defined by the administration of a second treatment on the lesion. Retreatment was decided by an experienced multidisciplinary tumor board composed of neurosurgeon oncologists, radiation oncologists and medical oncologists on a case-to-case basis, as there is currently no gold standard outside of surgical pathology to distinguish true tumoral progression from pseudoprogression or radiation necrosis. Since all patients in the cohort were in active treatment, and patients who only received supportive care were excluded, all retreated lesions were considered progressive, whereas all lesions that were not treated were considered to be responding, stable or showing non-tumoral progression, which encompassed pseudoprogression and radiation necrosis, in case of increased size. The multidisciplinary tumor board did not formally use RANO-BM criteria in their decision-making process; rather, they based their decision on diameter changes, surrounding FLAIR changes, enhancement pattern, lesion nodularity, T1–T2 lesion ratio and the timing of diameter changes relative to the SRS procedure [8]. Advanced MRI and PET imaging were not routinely used by our tumor board. No changes were made by our tumor board in metastases assessment during the study period.

RANO-BM response criteria were calculated retrospectively at each follow-up for every metastasis, as proposed in the original RANO-BM study for targeted metastases. CNS local progression-free survival was used as the endpoint. Complete response was defined as the complete disappearance of the metastasis; partial response was defined as decrease in diameter superior to 30%; stable disease was defined as decrease in diameter inferior to 30% or an increase in diameter of less than 20%; and progressive disease was defined as an increase in diameter of more than 20% from their nadir [4]. Corticosteroid intake and clinical status were not considered in this study, as our analyses were conducted on a metastasis-only level. Neurological symptoms and steroid dependence may be caused by concurrent metastases, unrelated to the evaluated metastasis, which would impact our assessment regarding progression or response. The RANO-BM score at last follow-up was used to evaluate the sensitivity, specificity, predictive positive value and predictive negative value of the RANO-BM response criteria relative to the decision of the multidisciplinary tumor board. A true positive corresponded to a metastasis which was retreated by SRS with a RANO-BM score corresponding to progressive disease. A false negative corresponded to a metastasis which was retreated by SRS with a RANO-BM score corresponding to complete response, partial response or stable disease. A true negative corresponded to a metastasis which was not retreated with a RANO-BM score corresponding to non-progressive disease. A false positive corresponded to a metastasis which was not retreated with a RANO-BM score corresponding to progressive disease. Non-tumoral progression was defined as a non-retreated increase in diameter attributed to pseudoprogression or radiation necrosis. RANO-BM score at last follow-up could therefore show non-progression based on the tumor evolution, irrespective of previous assessments. Non-tumoral progression rates were also evaluated after the second SRS treatment.

### 2.2. Statistical Analysis

Analyses were performed at patient-level for cohort demographics and at metastasis-level for RANO-BM criteria, non-tumoral progression and time delays. A receiver operating characteristic (ROC) curve was used to perform sensitivity analyses to evaluate RANO-BM’s current thresholds in diameter changes. A Kaplan–Meier estimator was used to evaluate non-tumoral progression and tumoral progression and compared using a Log-Rank test. Statistical significance was defined at *p* < 0.05. Statistical analyses were performed using IBM SPSS Statistics, version 25 (IBM, Armonk, New York, NY, USA) and figures were rendered using SPSS Statistics, version 25.

## 3. Results

### 3.1. Cohort Demographics

**Figure 1:**
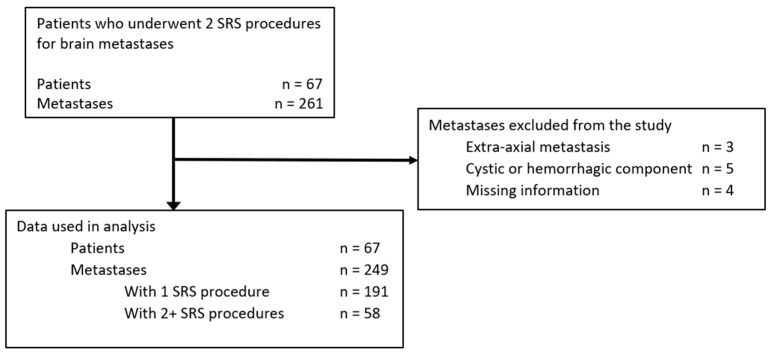
Sixty-seven patients underwent two SRS procedures for the relapse of a previously treated metastasis or for progression of a different untreated metastasis. Fifty-eight metastases were treated twice with SRS.

The patient selection process is detailed in Figure 1. Between 2007 and 2016, 67 patients underwent at least two SRS procedures for intracerebral metastases at the Centre Hospitalier Universitaire de Sherbrooke. Cohort demographics are presented in Table 1. Briefly, the median age at diagnosis of brain metastases was 53 years, ranging from 27 to 79 years, with 66% of patients being females. These patients had a total of 249 metastases which respected the inclusion criteria, with a median of 4 metastases per patient. Primary cancer histology was non-small cell lung cancer (NSCLC) in 57% of cases, breast cancer in 19%, melanoma in 9%, colorectal cancer in 7%, small cell lung cancer (SCLC) in 3%, thyroid cancer in 2%, renal cancer in 2% and unknown primary in 2%.

### 3.2. Metastases Characteristics and Outcomes

**Figure 2:**
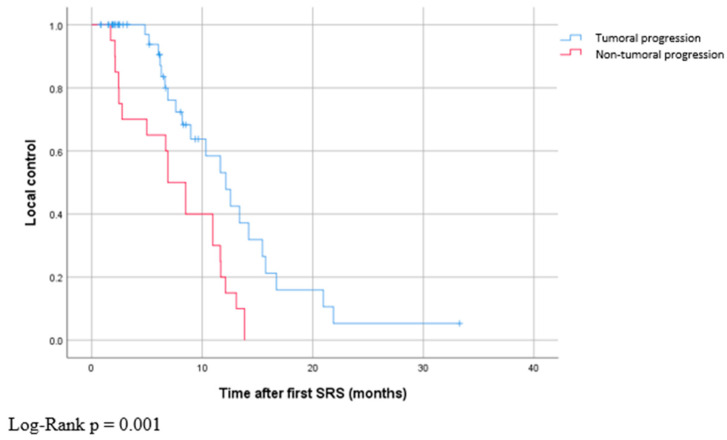
Time to progression in metastases with tumoral and non-tumoral progression.

As shown in Table 2, the median initial maximal diameter of intracerebral metastases in our cohort was 5.8 mm, ranging from 1.4 mm to 27.3 mm. The metastases were located in the cerebrum in 83% of cases, in the cerebellum in 16%, and in the brainstem in 1%. Regarding outcomes, the Kaplan–Meier estimator of local control is presented in Figure 2. Tumor progression was observed in 23% of metastases, with a median time between both SRS procedures of 12.1 months, ranging from 0.8 months to 33.3 months. Non-tumoral progression was observed in 8% of cases, with a median time of 6.9 months, ranging from 1.7 to 13.8 months. Non-tumoral progression occurred significantly earlier than tumoral progression (Log-Rank *p* = 0.001). No statistical differences in terms of diameter increases were noted between non-tumoral progression and real tumor progression (*p* = 0.41). Non-tumoral progression after second SRS was observed in 4% of cases.

### 3.3. RANO-BM Response Criteria Verification

RANO-BM response criteria at the last follow-up were stratified in two categories for each metastasis: progression (which warranted retreatment), and non-progression (which included complete response, partial response, stable disease and non-tumoral progression). In this series of 58 metastases which needed retreatment based on our multidisciplinary tumor board assessment of progressive disease, only 38% showed concordance with RANO-BM criteria and corresponded to true positives. In the 191 metastases which did not undergo retreatment, RANO-BM criteria confirmed non-progression in 95%, which corresponded to true negatives. RANO-BM criteria for progression, as currently defined by the RANO-BM group by an increase in diameter of 20% relative to the nadir, and of at least 5 mm, had a sensitivity of 38%, a specificity of 95%, a positive predictive value of 71% and a negative predictive value of 84% when applied to our cohort, as presented in Table 3.

**Figure 3:**
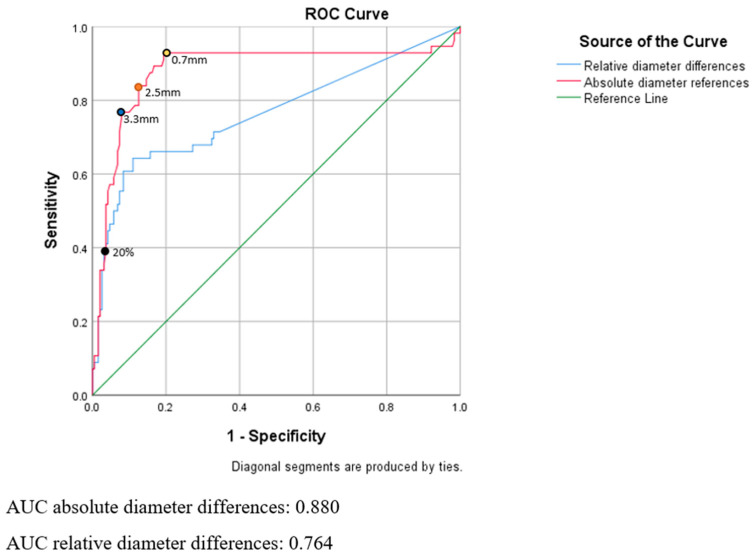
ROC curve of stratified RANO-BM score.

The ROC curve of the stratified RANO-BM criteria is illustrated in Figure 3. The blue curve represents the changes in sensitivity and sensibility caused by relative percentage increases between metastases diameter at different times during follow-up, such as the 20% increase in diameter in the original RANO-BM definition shown by the black point on Figure 3. The red curve represents the changes in sensitivity and sensibility caused by absolute increases in metastases diameter at different times during follow-up. The use of absolute increases as thresholds for stratified RANO-BM criteria instead of relative percentage increases achieves better test efficiency, with better global sensitivity and specificity in our series. The Area Under Curve corresponding to the ROC curve of absolute increases in metastases diameter is 0.880, compared to the ROC curve of relative increases in metastases diameter, with an Area Under Curve of 0.764.

Stratified RANO-BM criteria were recalculated by using as a threshold an absolute diameter increase of 2.5 mm relative to the nadir, which yielded the best efficiency on the ROC curve, as shown by the orange point on Figure 3. Using this modified threshold, RANO-BM criteria have a sensitivity of 83%, a specificity of 87%, a positive predictive value of 67% and a negative predictive value of 94%, as shown in Table 3.

Two other threshold values were extracted from the ROC curve in order to maximize either sensitivity or specificity. A threshold value of 0.7 mm, as shown by the blue point on Figure 3, yielded the highest sensitivity, at 93% with a specificity of 81%, a positive predictive value of 60% and a negative predictive value of 98%. A threshold value of 3.3 mm, as shown by the yellow point on Figure 3, yielded the highest specificity, at 92% with a sensitivity of 77%, a positive predictive value of 75% and a negative predictive value of 93%.

## 4. Discussion

### 4.1. RANO-BM Response Criteria and Potential Changes

This study is the first to externally evaluate the RANO-BM response criteria thresholds on diameter. In our cohort of patients who underwent at least two SRS procedures for intracerebral metastases, the original criteria showed high specificity at the cost of lower sensitivity. RANO-BM response criteria were designed to provide a more uniform classification of intracerebral metastases in a research setting [4], in which specificity should be prioritized due to the importance of identifying non-progression, especially during clinical trials. However, our results show that by using absolute diameter differences instead of relative diameter differences, sensitivity could be significantly improved with little impact on specificity. This finding could be explained by the fact that for smaller metastases, the RANO-BM criteria would underestimate progression. The current criteria define progression as an increase in diameter of 20% relative to nadir, and of at least 5 mm. In smaller brain metastases, such as those treated by SRS, the 20% relative diameter increase would not be used to evaluate progression. Considering our median metastasis diameter of 5.8 mm, a 20% increase would represent an increase of 1.2 mm, which is markedly inferior to the minimum absolute diameter increase of 5 mm recommended by the original description. Furthermore, a 5 mm increase relative to a 5.8 mm median diameter would represent an 86% diameter increase, which significantly lowers sensitivity as most multidisciplinary tumor boards would have considered that progression at lower diameter increases thresholds.

We propose considering a 2.5 mm absolute diameter increase as progression as it significantly improves sensitivity while only slightly reducing specificity, which could potentially be useful in a clinical setting. For situations in which specificity should be prioritized, such as clinical trials evaluating the impact of systemic therapies on metastases diameter, an absolute diameter increase of 3.3 mm should be considered as progression based on our data. The 2.5 mm absolute diameter threshold yields a specificity only 8% lower than in the original RANO-BM description, but with a sensitivity that is 45% higher. For situations in which sensitivity should be prioritized, such as an incidence study on recurring metastases, a threshold of 0.7 mm could be used. With recent advances in T1 resolution, especially in research due to more widespread use of 3T MRI [9] and even 7T MRI [10] in certain centers, inframillimetric diameter changes should be considered as significant rather than related to partial volume effects.

### 4.2. Non-Tumoral Progression vs. Tumoral Progression

Pseudoprogression is an important concern when evaluating diameter increases in neuro-oncology. Targeted therapies, such as immune checkpoint inhibitors frequently used in metastatic NSCLC and melanoma, which were the primary cancer in 76% of patients in this series, can result in temporary increases in size secondary to lymphocyte infiltration [11]. However, this should be minimized in our study by the fact that during the study period (from 2007 to 2016), such therapies were still considered experimental and were not as widely used as they are now. Local control could therefore be mainly attributed to SRS in our cohort. SRS can also lead to radiation-induced changes such as radiation necrosis, which is indistinguishable from tumoral progression on conventional imaging [12]. Our data show that the RANO-BM response criteria score can equally assess non-tumoral progression and tumoral progression. While imaging changes are not reliably used to distinguish both types of progression, our survival analysis shows that detectable non-tumoral progression occurs significantly earlier than detectable tumoral progression. This finding, in line with previous reports of pseudoprogression’s earlier onset [13], could possibly facilitate differentiating both progression types based on the time of onset; progression during the first few months would be more likely caused by non-tumoral progression, while progression occurring after more than a year would be more suggestive of tumoral progression warranting retreatment. Our results also show that diameter changes should not be used to differentiate non-tumoral progression from tumoral progression, as it was not shown to be a differentiating factor. Non-tumoral progression was defined in this study as a non-retreated increase in diameter followed by an eventual decrease in diameter before the last follow-up. As non-tumoral progression is not a definite diagnosis in the absence of a biopsy or neurosurgical resection, we cannot exclude that lesions considered by our tumor board as tumoral progression might have been pseudoprogression had we waited before commencing retreatment. However, our low non-tumoral progression rate of 4% after the second SRS suggests that our tumor board efficiently identified real tumoral progression. Retreatment of non-tumoral progression would have yielded higher rates of non-tumoral progression after the second SRS.

### 4.3. Limitations

This study is limited by its retrospective nature. While all patients were in an active treatment period, therapeutic intensity could vary depending on systemic disease and its related prognosis, neurological symptoms, comorbidities and patient choice. This could impact our data, as our gold standard for tumoral progression in this study was the decision made by our multidisciplinary tumor board to repeat SRS. However, since at least one metastasis was retreated by SRS for each patient, we can safely assume that systemic disease and patient intention were uniform throughout the active treatment period and that significant progression was retreated as per neuro-oncology guidelines. Follow-up timeframes were also variable in this study, which made time-related analyses—such as the delay between the first SRS and RANO-BM criteria showing progression or the delay between RANO-BM criteria showing progression and the second SRS—impossible, as too many confounding factors could affect our results. These limitations could be addressed by a prospective study with clearly defined follow-up times. The use of our tumor board as the gold standard for tumoral progression is also a limitation of this study, as the definition of tumoral progression could vary between healthcare centers for equivocal diameter increases. However, a previous study from our group in a similar cohort showed that our management of tumoral progression seemed adequate, as it resulted in favorable outcomes such as long-term local control and improved clinical status, with low rates of radiation-induced complications such as edema or pseudoprogression [14]. A similar verification study could also be performed by other centers to evaluate the impact of local treatment preferences on RANO-BM criteria thresholds. Furthermore, while non-tumoral progression is indistinguishable from tumoral progression with conventional imaging, the use of advanced imaging techniques such as MR spectroscopy, diffusion-weighted or perfusion MR imaging and amino acid TEP scans could be considered to evaluate non-tumoral progression more adequately in a similar cohort [15]. 

### 4.4. Impact of Our Study on the Current Clinical Management of Recurrent Brain Metastases

SRS is now the single most-used management modality for brain metastases and is increasingly repeated in the setting of locally recurrent disease. As is the case for any normal tissue sparing radiation technique, the smaller the target, the higher the likelihood of achieving this goal. We have demonstrated in this study that using an absolute threshold of 2.5 mm increase in diameter to confirm local progression improves sensitivity compared to the use of the RANO-BM criteria, which could accelerate confirmation of recurrent disease. This would allow repeat SRS to be performed earlier on a smaller target, thus limiting the radiation received by the normal brain parenchyma, which has the potential to reduce the inherent risks of reirradiation, to the benefit of the patient.

## 5. Conclusions

The RANO-BM response criteria current thresholds seem to unreliably identify clinically relevant tumor progression, as shown by their low sensitivity in our cohort. Using absolute diameter thresholds instead of relative diameter increases might be a useful tool to guide management of brain metastases that show increased size after SRS, as was demonstrated in our cohort by the improvement in sensitivity, despite the low impact on specificity.

## Figures and Tables

**Table 1:** Cohort demographics.

		*n* (%) or Median (Range)
Median age at diagnosis of brain metastases		53 (27–79)
Median number of brain metastases per patient		4 (1–22)
Sex	Female	44 (66%)
	Male	23 (34%)
Primary cancer histology	NSCLC	38 (57%)
	Breast	13 (19%)
	Melanoma	6 (9%)
	Colorectal	5 (7%)
	SCLC	2 (3%)
	Thyroid	1 (2%)
	Renal	1 (2%)
	Unknown	1 (2%)
Primary cancer surgery before SRS	No	29 (44%)
	Partial resection	8 (12%)
	Complete resection	29 (44%)
Primary cancer radiation before SRS	No	41 (61%)
	Fractionated radiotherapy	25 (37%)
	SRS	1 (2%)
Primary cancer systemic treatment before SRS	No	13 (19%)
	Chemotherapy	48 (72%)
	Targeted therapy	6 (9%)

**Table 2:** Metastases characteristics and outcomes.

		*n* (%) or Median (Range)
Metastasis initial maximal diameter (mm)		5.8 (1.4–27.3)
Metastasis location	Cerebrum	207 (83%)
	Cerebellum	41 (16%)
	Brainstem	3 (1%)
Time between first SRS and second SRS (months)		13.5 (2.8–47)
Metastasis tumor progression		58 (23%)
Metastasis non-tumoral progression		20 (8%)
Median time to non-tumoral progression		6.9 (1.7–13.8)
Median time to tumor progression		12.1 (0.8–33.3)
Median-diameter increase in non-tumoral progression		11.5 (5.8–25.9)
Median-diameter increase in tumoral progression		5.7 (1.6–26.2)
WBRT after first SRS for tumor progression		13 (5%)

**Table 3:** RANO-BM score verification at last follow-up. A total of 58 out of 249 metastases (23%) showed tumoral progression after the first SRS treatment, warranting retreatment as per the tumor board evaluation.

Threshold Values	20%	0.7 mm	2.5 mm	3.3 mm
True positives	22	54	48	45
False negatives	36	4	10	13
True negatives	182	155	167	176
False positives	9	36	24	15
Sensitivity	38%	93%	83%	77%
Specificity	95%	81%	87%	92%
Positive predictive value	71%	60%	67%	75%
Negative predictive value	84%	98%	94%	93%

## Data Availability

The data presented in this study are available on request from the corresponding author.
